# Supplementary material for: The effects of a 4-week mesocycle of barbell back squat or barbell hip thrust strength training upon isolated lumbar extension strength
Source: PeerJ. 2019 Jul 26;7:e7337. doi: 10.7717/peerj.7337 (PMC6662562; doi:10.7717/peerj.7337)
Supplement: Supplemental Information 2 [file peerj-07-7337-s002.pdf]

A more detailed explanation of strength index (SI) as the area under the curve of a force graph:

Maximal isometric testing was performed at 7 joint angles (0˚, 12˚, 24˚, 36˚, 48˚, 60˚ and 72˚ of extension; data was collected in ft.lbs)

Based on hypothetical values the data (table 1) and strength curve (figure 1) would look something like this.

Table 1.

Angle	0	12	24	36	48	60	72
Force (ft.lbs)	190	200	210	220	230	240	250

Figure 1.

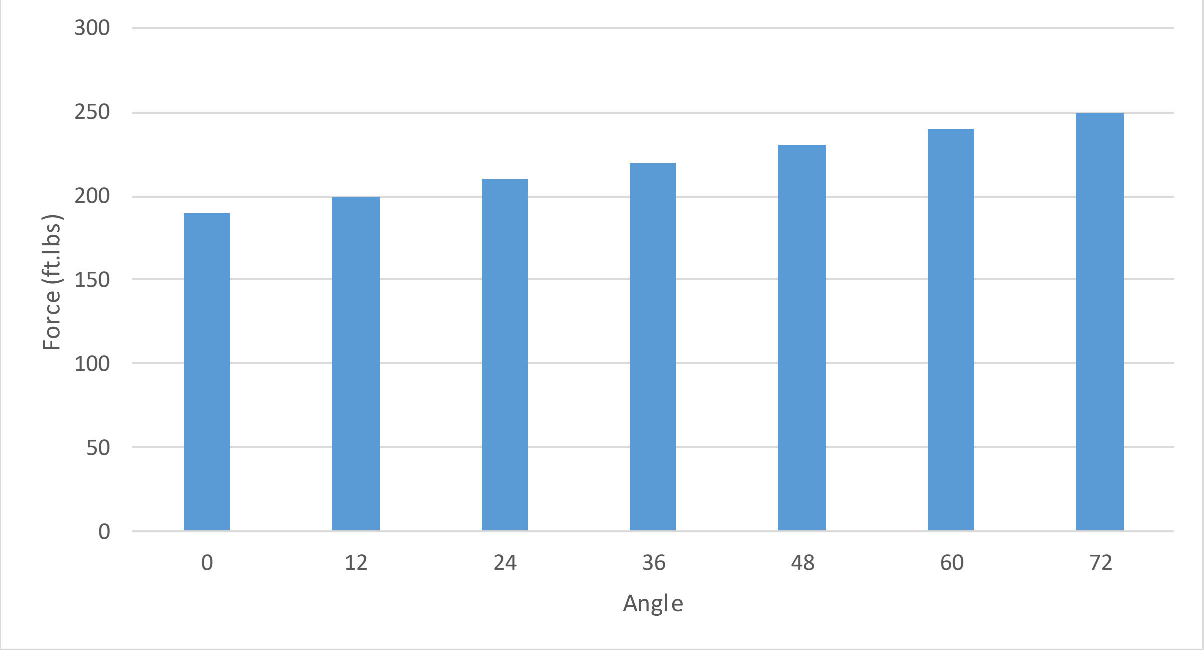

Using the following formula (Figure 2) to calculate the area of a trapezoid the following values are calculated for the respective areas of the curve (table 2), depicted in figures 3 and 4.

Figure 2

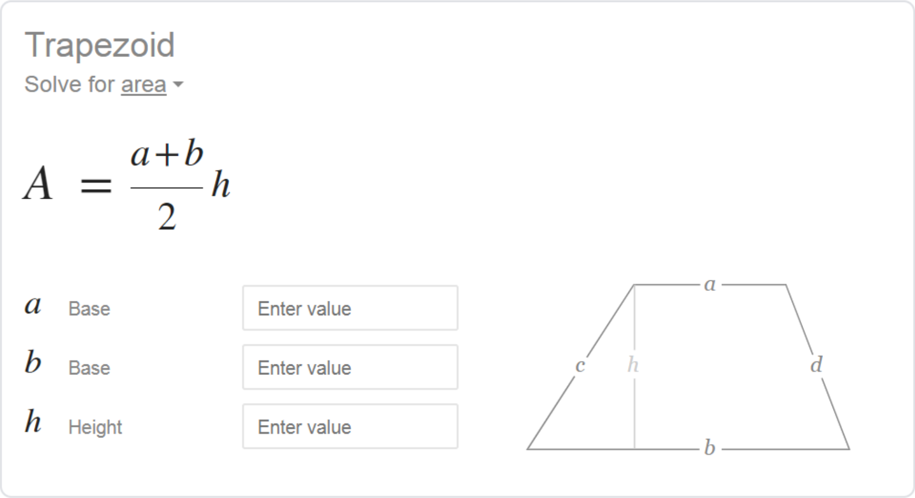

Table 2

Strength index for area						
0-12deg (a)	12-24deg (b)	24-36deg (c)	36-48deg (d)	48-60deg (e)	60-72deg (f)	Total (g)
2340	2460	2580	2700	2820	2940	15840

Figure 3

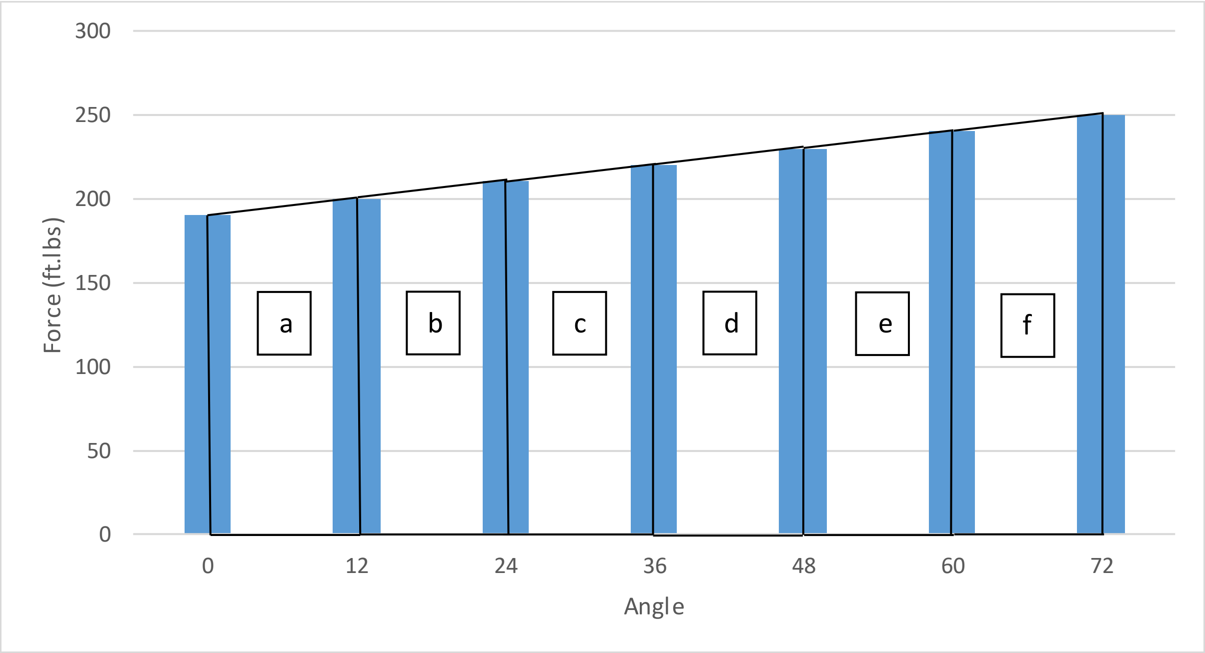

Figure 4

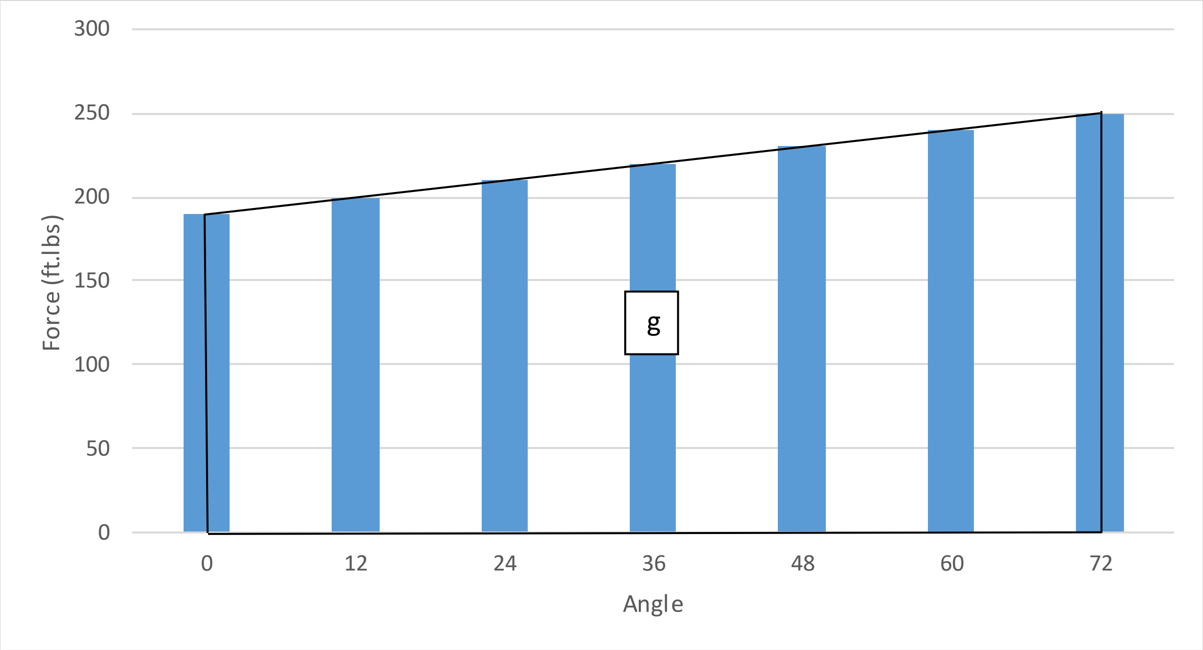

Our MedX Lumbar extension machine produces this data using these methods giving us the total value of 15840ft.lbs·degrees. We convert the data to Newton metres by multiplying by 1.3556. Giving us a final strength index for the area under the curve (e.g. "g") of 21472Nm·degrees. The strength index is an integral and thus expressed as Nm·degrees.
